# First case of primary intraocular natural killer t-cell lymphoma

**DOI:** 10.1186/s12886-015-0158-0

**Published:** 2015-11-19

**Authors:** Kazuichi Maruyama, Hiroshi Kunikata, Sunao Sugita, Manabu Mochizuki, Ryo Ichinohasama, Toru Nakazawa

**Affiliations:** Department of Ophthalmology and Visual Science, Tohoku University Graduate School of Medicine, Sendai, Japan; RIKEN Center for Development Biology, Kobe, Japan; Department of Ophthalmology, Tokyo Dental and Medical University, Tokyo, Japan; Department of Hematopathology, Tohoku University Graduate School of Medicine, Sendai, Japan

**Keywords:** Natural killer cell, Vitreous sample, Flow cytometry, Cytology, Epstein-Barr virus

## Abstract

**Background:**

Natural killer cell tumors can be broadly divided by origin into mature-cell and progenitor-cell types. The invasion of nasal-origin natural killer cells into the ophthalmologic field is sometimes observed in patients, but primary ocular natural killer cell tumors are a rare occurrence.

**Case presentation:**

A 66 year-old woman without any systemic disease presented with blurred vision due to a severe vitreous opacity in the right eye. Flow cytometric analysis of the vitreous fluid suggested a natural killer cell tumor. Moreover, cytologic examination of vitreal and retinal specimens revealed the infiltration of a natural killer cell tumor, while PCR and immunocytochemistry revealed Epstein-Barr virus infection. The results of a gene rearrangement analysis were positive for IGH, while TCR beta chains were all negative. We examined the patient with whole-body magnetic resonance imaging and positron emission tomography, and performed a bone marrow examination. These examinations returned no abnormal results.

**Conclusion:**

Thorough analysis of vitreal samples is essential when performing vitrectomies for vitreous opacities of unknown cause. Flow cytometric, cytologic, and PCR analysis of vitreal and retinal samples may reveal the presence and cause of severe illness.

## Background

Natural killer-T (NK-T) cell lymphoma, previously categorized as a lethal granuloma, is now a definitive diagnostic entity in the World Health Organization lymphoma classification [1]. Two main categories of NK-T cell lymphoma are recognized: aggressive NK cell leukemia and extranodal NK-T cell lymphoma, nasal type. Most NK-T cell lymphomas in ocular tissue are expansions or invasions from nasal-type tumors. A few cases of NK-T cell lymphomas that expanded to the ocular tissue have been reported [2], but there have been no reports of primary intraocular NK-T cell lymphomas. To diagnose this very malignant type of tumor, it is important to evaluate cell surface markers and confirm the presence of Epstein-Barr virus infection. Unlike large granular lymphocyte T-cells, this type of tumor is negative for CD3 surface markers, but positive for cytoplasmic CD3 epsilon and CD56 surface markers.

**Fig. 1:**
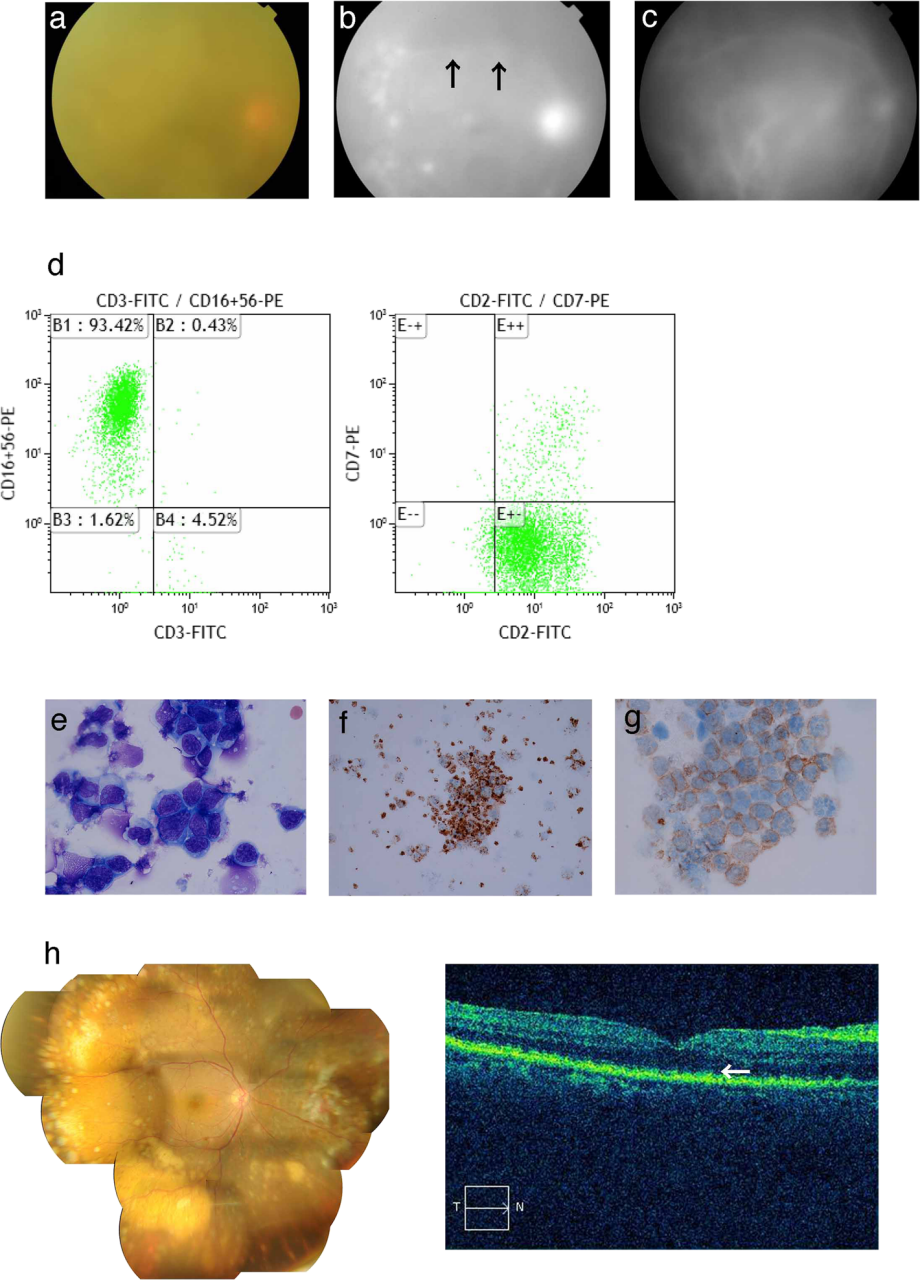
Photograph of the posterior segment showing a severe vitreous opacity with a reddish disc (**a**). Fluorescence angiography image showing leakage (indicated by the arrow) (**b**). Indocyanine green angiography image (**c**). Flow cytometry data indicating that CD2- and CD56-positive cells were present in vitreous specimens (**d**). Cell cytology image indicating abnormally shaped nuclei after Giemsa staining (**e**). TIA-1 (**f**) and Cyclin D3; CyCD3 (**g**) were also found. Photograph of the posterior segment after irradiation and chemotherapy (**h**), Multiple white or cream-colored lesions were observed. OCT image of the macular area (**i**) showing disease lesions at the level of the retinal pigment epithelial layer (indicated by the *arrow*)

**Fig. 2:**
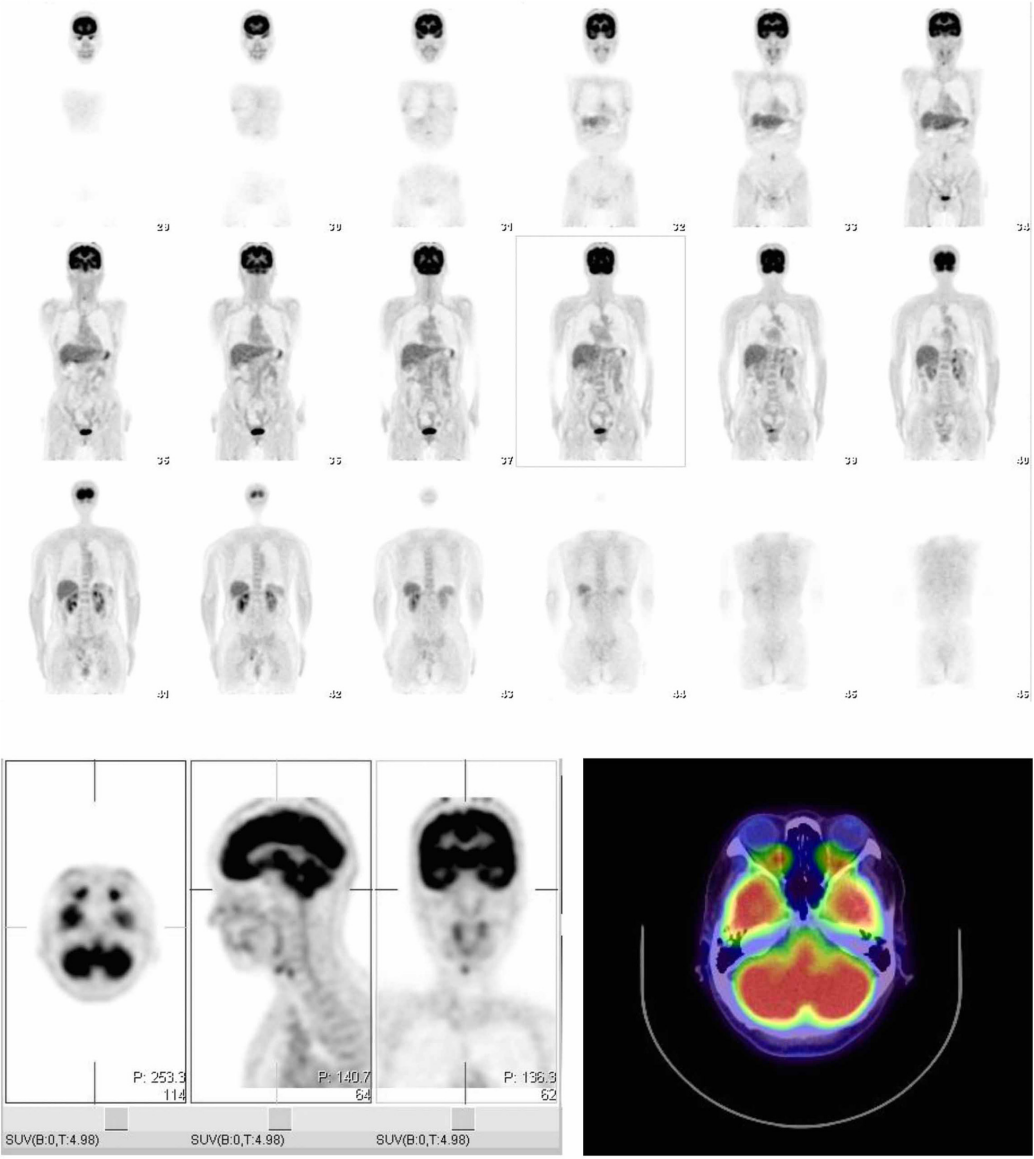
Positron emission tomography images. No extra-ocular tumor was found

## Case presentation

On March 14, 2014, a 66-year-old woman visited our university hospital with long-term blurred vision due to a prolonged, severe vitreous opacity. The patient had been undergoing treatment with oral and local steroids since November 2013. The symptoms had temporarily improved after the steroid treatment, but there was no permanent, significant improvement. Thus, the patient was referred to us in order to uncover the cause of the vitreous opacity.

During our examination, we found that the patient’s visual acuity was hand motion 30 cm oculus dexter (OD), and 20/20 oculus sinister (OS). The left eye of the patient was completely unaffected during the course of clinical observation. Intraocular pressure was 12 mmHg OD and 15 mmHg OS. Slit lamp examination did not reveal inflammation in the anterior chamber, except for a slight conjunctival injection. In contrast to the anterior segment, a severe vitreous opacity and the infiltration of relatively small cells was found in the posterior segment OD (Fig. 1). However, there was only minor vasclutis, with no evidence of vascular leakage or exudate in the retina caused by the vitreous opacity. The opacity did not respond to steroid treatment.

A tentative diagnosis of primary intraocular lymphoma was made. Vitreous collection was then performed with 25-gauge microincision vitrectomy, and a retinal biopsy was taken. This study was approved by the Institutional Review Board of the Tohoku University Graduate School of Medicine. All experimental procedures were conducted in accordance with the tenets set forth in the Declaration of Helsinki. The study was registered in the University Hospital Medical Information Network (UMIN) Clinical Trial Registry (CTR) (UMIN000004980). During the operation, multiple white or cream-colored lesions were found in the retina. Vitreous specimens were collected with or without BSS perfusion. The specimens without perfusion were used for multiplex polymerase chain reaction (PCR), in order to detect possible infections. The genomic DNA of bacteria, fungi, parasites and viruses in the vitreous were examined with comprehensive PCR [3]. The samples with BSS perfusion were processed for flow cytometry, cytology and gene rearrangement with the Registration Examination and Analysis Description (READ) system [4].

Findings from the flow cytometry analysis revealed the infiltration of cells positive for CD45, CD2 and CD56, but not CD4, CD8, CD19 or CD30 (Fig. 1). Furthermore, the immunocytochemical analysis revealed that more than 90% of cells positive for CD2, CD56, CyCD3 and T-cell intracytoplasmic antigen (TIA) 1–1 (Fig. 1) were also positive for Ki67. A histological examination of the retina indicated that the tumor was malignant. Additionally, the cells infiltrating the vitreous had abnormally shaped nuclei, and evidence of apoptosis was found in the retina between the intra-lamellar membrane and the inner plexiform layer. Both PCR analysis and in situ hybridization revealed that Epstein Bar virus DNA and Epstein Bar virus-encoded small RNA (EBER) were present in the vitreous specimens. Moreover, the results of a gene rearrangement analysis were positive for the immunoglobulin heavy locus (IGH) and T-cell receptor (TCR) beta chains, especially the TCR beta chain, while the TCR alpha, beta, gamma and delta chains were all negative. This result indicated a clonal, abnormal chromosome. Next, we examined the patient with whole body magnetic resonance imaging and positron emission tomography (Fig. 2), and performed a bone marrow examination. These examinations returned no abnormal results. Finally, after consideration of all the clinical examinations, including the histological analysis, a diagnosis was made of primary intraocular NK tumor cell lymphoma (NKTL). Therefore, we decided to treat the patient with the intraocular injection of methotolexate following irradiation and 2 courses of SMILE (dexamethasone, methotrexate, ifosfamide, L-asparaginase, and etoposide) chemotherapy.

## Conclusion

This is the first report of a case in which primary intraocular NKTL was diagnosed from vitreous specimens. NKTL is usually found in intraocular tissue as an invasion of extranodal NKTL, particularly the nasal type [5]. However, our patient presented with only an intraocular tumor, without any affected extra-ocular tissue. NKTL is known as a lethal midline granuloma, and is now a diagnostic entity in the World Heath Organization lymphoma classification [1]. Moreover, NKTL is known as a rapidly progressing disease, with a short patient survival time after diagnosis [6]. Therefore, prompt and precise diagnosis is vital for NKTL treatment. Previous reports have indicated that intraocular NKTL also carries the risk of leptomeningeal or central nervous system dissemination. Our patient received radiation therapy with systemic chemotherapy soon after diagnosis, and fortunately, has not had any central nervous system dissemination in recent examinations.

Routine examination with our READ system promises to allow the precise diagnosis of primary intraocular NKTL and its discovery at an early stage. While the cause of vitreous opacity in NKTL cannot be determined with certainty from the single case described here, our findings show that diagnosis must be based on the analysis of vitreous specimens, in addition to flow cytometry [7], cytology and PCR analysis.

## Consent

Written informed consent was obtained from the patient for publication of this case report and any accompanying images. A copy of the written consent is available for review by the editor of this journal.

## Abbreviations

NK-T: Natural killer T cell
OD: Oculus Dexter
OS: Ocular Sinister
UMIN: University Hospital Medical Information Network
CTR: Clinical Trial Registry
PCR: Polymerase chain reaction
READ: Registration Examination and Analysis Description
TIA: T-cell intracytoplasmic antigen
EBER: Epstein Bar virus encoded small RNA
IGH: Immunoglobulin heavy locus
TCR: T cell receptor
NKTL: NK tumor cell lymphoma
CyCD3: Cyclin D3
